# Chemical Profile and Antibacterial Effect of *Pimenta dioica* Essential Oil Against *Ralstonia solanacearum* Race 2 Causing Moko Disease on Banana Crop

**DOI:** 10.3390/plants15101515

**Published:** 2026-05-15

**Authors:** Luciano Martínez-Bolaños, Victor López-Martínez, Cristian Nava-Díaz, Artemio Pérez-López, Syl Soledad Martínez-Bolaños, Gilberto Manzo-Sánchez, Moisés Roberto Vallejo-Pérez, Misael Martínez-Bolaños, Mario Orozco-Santos, Carlos Hugo Avendaño-Arrazate

**Affiliations:** 1 Centro Académico Regional sede Oaxaca, Ingeniería en Agricultura Sostenible, Posgrado en Protección Vegetal, IIPPV, Fitosanidad Tropical, Universidad Autónoma Chapingo, Zimatlán, Oaxaca 71200, Mexico; 2 Postgrado en Ciencias Agropecuarias y Desarrollo Rural, Facultad de Ciencias Agropecuarias, Universidad Autónoma del Estado de Morelos, Cuernavaca 62209, Mexico; victor.lopez@uaem.mx; 3 Fitosanidad-Fitopatología, Colegio de Postgraduados, Texcoco 56230, Mexico; cnava@colpos.mx; 4 Posgrado en Ciencia y Tecnología Agroalimentaria, Departamento de Ingeniería Agroindustrial, Universidad Autónoma Chapingo, Texcoco 56230, Mexico; aperezl.dia@gmail.com; 5 Departamento de Ingeniería Agroindustrial, Universidad Autónoma Chapingo, Texcoco 56230, Mexico; sylsoledad@gmail.com; 6 Facultad de Ciencias Biológicas y Agropecuarias, Universidad de Colima, Colima 28930, Mexico; gmanzo@ucol.mx; 7 Coordinación para la Innovación y Aplicación de la Ciencia y Tecnología, Universidad Autónoma de San Luis Potosí, San Luis Potosí 78210, Mexico; vallejo.pmr@gmail.com; 8 Campo Experimental Rosario Izapa, Instituto Nacional de Investigaciones Forestales, Agrícolas y Pecuarias, Tuxtla Chico 30870, Mexico; avendano.carlos@inifap.gob.mx; 9 Campo Experimental Tecomán, Instituto Nacional de Investigaciones Forestales, Agrícolas y Pecuarias, Colima 28930, Mexico; orozco_santos@yahoo.com

**Keywords:** *Pimenta dioica*, bacterial wilt disease, sustainable management, eugenol, bioessential oils

## Abstract

Moko disease (*Ralstonia solanacearum* race 2) is one of the most destructive bacterial diseases affecting bananas and plantains worldwide. The pathogen infects banana plants, causing yellowing and wilting of younger leaves, and plant death. Disease management remains challenging due to the pathogen’s aggressiveness, rapid dissemination, and limited availability of effective control products. The aim of this study was to determine the chemical composition of the *Pimenta dioica* essential oil (PDEO) obtained by hydro-distillation and to evaluate its antibacterial activity against *R. solanacearum* race 2. Gas chromatography-mass spectrometry (GC-MS) analysis identified 19 compounds in the essential oil. Eugenol (72.6%), was the predominant component, followed by caryophyllene (6.13%) and Beta-Myrcene (4.17%). In vitro assays demonstrated complete inhibition of bacterial growth at 500 µL L^−1^. Probit analysis estimated the minimum inhibitory concentration 95% (MIC_95_) value 297.6 µL L^−1^. In plants evaluation using banana vitroplants showed that PDEO at 500 µL L^−1^ effectively reduced disease severity and prevented internal corm discoloration without causing phytotoxic effects. These findings demonstrate the strong antibacterial activity of *P. dioica* essential oil against *R. solanacearum* race 2 and highlight its potential as a natural alternative for the management of Moko disease in banana production systems.

## 1. Introduction

Bananas (*Musa acuminata* Colla AAA group) and plantains (*Musa balbisiana* Colla ABB group) are among the most important fruit crops worldwide due to their nutritional value and their social and economic impact. These crops are cultivated in 132 countries, covering approximately 5,940,159 ha, with a total production of 1,351,112,326 tons [1]. In Mexico, 87,393.92 ha are dedicated to banana and plantain cultivation, producing 2,670,290.89 tons annually [2].

Bacterial wilt disease or Moko disease of banana and plantain, caused by the betaproteobacteria *Ralstonia solanacearum* Phylotype II race 2, is one of the most destructive bacterial diseases affecting Musaceae worldwide [3,4]. The pathogen has been reported in commercial banana plantations in three continents: (a) Africa: Ethiopia, Libya, Nigeria and Senegal; (b) Asia: India, Indonesia, Malaysia, Philippines, Thailand, and Vietnam [5]; (c) America: Belize, Brazil [6], Colombia, Costa Rica, Ecuador [7], El Salvador, Grenada, Guadeloupe, Guatemala, French Guiana, Honduras, Jamaica, Mexico, Nicaragua, Panama, Peru, Saint Vincent and the Grenadines, Surinam, Trinidad and Tobago, United States, and Venezuela [8]. *Ralstonia solanacearum* race 2 enters banana plants through natural apertures or wounds, and colonizes the xylem vessels, obstructing water and nutrient transport. This results in yellowing of young leaves, wilting and eventually leads to plant death. The pathogen spreads easily through insects, contaminated tools and planting materials in commercial plantations, as well as through infected propagative materials or contaminated soil on newly established fields. Flooding and irrigation systems also facilitate its dissemination. The bacteria can survive up to 18 months in high soil humidity and with amenable host plants [4,9,10]. The impact of Moko disease on plantains and bananas crops has led to development of molecular techniques for the diagnosis and early detection of *Ralstonia solanacearum* race 2 [11,12,13,14,15], studies on genetic and pathogenic diversity [16,17,18]; assessments climatic adaptability [19,20], investigations of plant defense-related compounds [21,22], and the development of epidemiological models of Moko disease [23].

Management of Moko disease is primarily focused on prevention and includes prophylactic and exclusion measures to reduce the risk of spreading to disease-free areas. These measures include quarantine restrictions, the use of vitroplants or certificated propagation material, and the cultivation of tolerant genotypes such as FHIA-21 [24]. In areas where the disease is already present, management is challenging due to the aggressiveness of the pathogen and its high dispersal capacity. Therefore, strategies aimed at limiting bacterial dissemination are required, including cultural practices, and physical or chemical treatments [4]. Chemical control measures include the application of copper-based products, quaternary ammonium salts, glycyrrhizic acid ammonium salt nanoparticles [25], glyphosate injection [26] and antibiotics. However, repeated chemical applications may promote resistance in plant pathogenic bacteria and increase production costs and environmental risks. Consequently, the development of sustainable alternatives for control strategies is a priority. Efforts have also focused on biological control approaches, including the use of secondary metabolites from *Bacillus* sp. [27]; bacteriophages [28,29]; and elicitors that act as resistance inducers [30]. Essential oils (EOs) are a natural mixture of volatile secondary metabolites, mainly terpenoids and oxygen derivatives, characterized by specific aromas depending on the plant species and extraction method [31]. EOs present great potential against plant pathogens, including fungi [32,33], nematodes [34], bacteria [35,36,37,38], insects, and mites [39]. Their lipophilic nature facilitates penetration into bacterial cells, altering the proportion of unsaturated fatty acids in the membrane, reducing membrane fluidity, and promoting the entry of compounds [40]. These alterations disrupt osmotic balance and cellular integrity, leading to membrane damage, cell wall lysis, shrinkage, deformation, leakage of lipid molecules and alkaline phosphatase (AKP), and ultimately bacterial death [41,42,43]. Moreover, essential oils (EOs) present a low risk of inducing microbial resistance due to their multiple mechanisms of action against plant pathogenic bacteria [44]. They are also generally considered safe for humans and the environment [42]. The antibacterial activity of essential oils has been reported for several plants, including *Trachyspermum ammi* L. [42], *Pelargonium graveolens* [45], *Thymus vulgaris* [46], *Artemisia argyi* [43], *Artemisia arborescens* [47], *Syzygium aromaticum* [48], *Cymbopogon flexuosus* and *C. winterianus* [49]. Additionally, various studies have demonstrated the antibacterial activity of essential oils and plant extracts against *R. solanacearum*, including those from *Tagetes patula* [50], *Cymbopogon citratus* [51], *Cyphostemma cyphopetalum* [52], *Cinnamomum zeylanicum* [53], *Pimenta racemosa* var *racemosa* [54] and *Mentha piperita* L. [55].

The objective of this study was to evaluate the antibacterial effect of *Pimenta dioica* essential oil (PDEO) against *Ralstonia solanacearum* race 2, and its contribution to the development of sustainable strategies for the integrated management of Moko disease in banana and plantain crops.

## 2. Results

### 2.1. Phytochemical Composition of Pimenta Essential Oil

**Figure 1:**
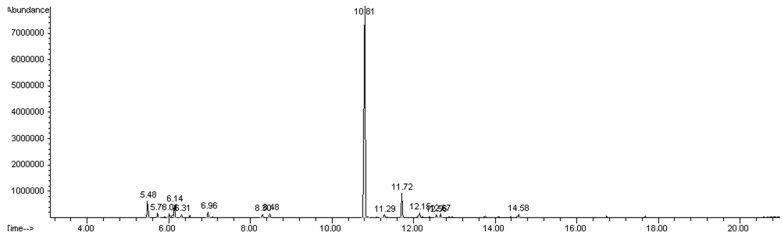
GC-MS chromatogram of essential oil from *Pimenta dioica*. The main peak represents eugenol.

For each extraction, 1.7–2.0 mL 50 g^−1^ yield of pimenta essential oil was obtained. GC-MS analysis of PDEO revealed a complex chemical profile, characterized by a diverse set of bioactive compounds. A total of 19 compounds were identified, representing 99.98% of the total oil composition. The major constituent was 3-Allyl-6-methoxyphenol (eugenol) accounting to 72.6% of the total composition. Other relevant compounds included caryophyllene (6.13%) and myrcene (4.17%) (Table 1, Figure 1). These results highlight the predominance of eugenol in PDEO and suggest that this compound, together with other minor constituents, may contribute to the biological activity of essential oil, supporting its potential application in plant disease management.

### 2.2. Antibacterial Activity Against Ralstonia solanacearum Race 2

**Figure 2:**
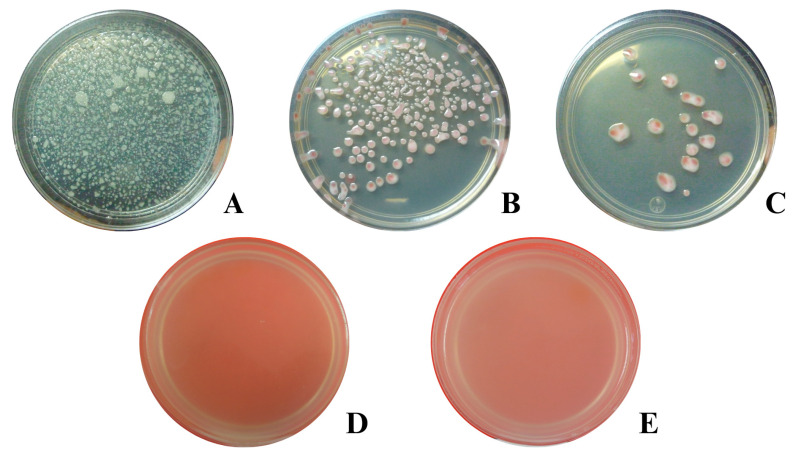
Antibacterial effect of pimenta (*Pimenta dioica*) essential oil against *R. solanacearum* race 2. (**A**) Control; (**B**) 100; (**C**) 300; (**D**) 500; (**E**) 1000 µL L^−1^.

The antibacterial activity of *P. dioica* was evaluated against *R. solanacearum* race 2. Bacterial growth decreased significantly with increasing concentration of PDEO. Colony-forming units (CFU) of *R. solanacearum* race 2 were completely inhibited at 500 µL L^−1^; however, at 100 and 300 µL L^−1^, bacterial colony growth was observed in a dose-dependent manner, with statistically significant differences (Tukey, 0.05) (Figure 2, Table 2). Probit analysis estimated the minimum inhibitory concentration required to inhibit 95% of bacterial growth (MIC_95_) at 297.6 µL L^−1^ (Table 3).

Analysis of variance of the results shown in the in vitro tests indicated that all evaluated concentrations had a statistically significant effect on the bacterial growth (*p* < 0.0001) (Table 2).

### 2.3. In Vivo Antimicrobial Activity of PDEO Against R. solanacearum Race 2

**Figure 3:**
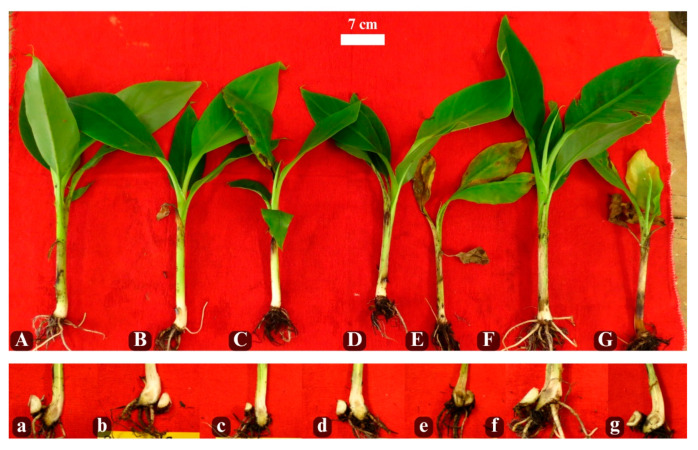
Banana vitroplants 42 days after inoculation with *R. solacearum* race 2 and treated with Pimenta (*Pimenta dioica*) essential oil. External symptoms (capital letter): **A**: 5000; **B**: 3000; **C**: 1000; **D**: 500; **E**: 300 µL L^−1^; **F**: Negative control; **G**: Positive control. Internal symptoms (lowercase letter): **a**: 5000; **b**: 3000; **c**: 1000; **d**: 500; **e**: 300 µL L^−1^; **f**: Negative control; **g**: Positive control.

Banana vitroplants inoculated with *R. solanacearum* race 2 and treated with different concentrations of PDEO exhibited dose-dependent differences in symptom severity. Forty-two days after inoculation, vitroplants treated with 300 µL L^−1^ of PDEO showed yellowing of upper leaves, and eventual plant death. Vitroplants treated with 500 µL L^−1^ showed no visible external symptoms. Untreated inoculated control vitroplants developed severe symptoms, including total wilting of upper leaves and plant death. Assessment of internal symptoms revealed that untreated inoculated plants exhibited dark discoloration throughout the rhizome. In contrast, vitroplants treated with PDEO showed reduced internal symptoms. Notably, vitroplants treated with concentrations ranging from 500 to 5000 µL L^−1^ displayed rhizomes without visible vascular discoloration (Figure 3).

## 3. Discussion

The successful and sustainable management of Moko disease relies on integrated strategies that include quarantine restrictions, preventive phytosanitary measures, breeding programs aimed at developing resistant banana genotypes, and systematic monitoring and eradication of infected plants. However, the persistence and rapid dissemination of *Ralstonia solanacearum* race 2 highlight the need for complementary and sustainable control strategies.

Essential oils (EOs) are complex mixtures of bioactive organic compounds with antimicrobial properties against a wide range of phytopathogens, including bacteria, fungi, and nematodes. Their traditional use in ethnobotany and plant protection is supported by their availability, biodegradability, and low environmental impact. Compared with conventional chemical pesticides, EOs represent promising alternatives due to their reduced persistence in the environment and lower risk of harmful residues in the food chain.

In the present study, GC-MS analysis revealed that eugenol was the predominant compound in *Pimenta dioica* essential oil, representing 72.6% of the total composition. This finding is consistent with previous reports identifying eugenol as the main constituent of *P. dioica* essential oil [56,57,58,59,60]. Reported concentrations vary depending on plant material and conditions, ranging from 59.8% in fresh and mature leaves [59] to 65.9–71.4% in other studies [61]. Such variation may be attributed to differences in plant tissue, environmental conditions, soil characteristics, elevation and water availability, harvest time, crop management, postharvest handling, and extraction methodology [62].

*P. dioica* essential oil has been documented for its antifungal activity [63,64]; however, our results revealed the antibacterial activity against *Ralstonia solanacearum* race 2. The antibacterial effect observed is attributed to the high eugenol content, a phenolic compound known to disrupt bacterial cell membranes, alter membrane permeability, and interfere with essential metabolic processes [65], including DNA, RNA, and protein synthesis [66].

Our results showed a clear concentration-dependent inhibition of bacterial growth. At 100 μL L^−1^, growth inhibition reached 68.9%, increasing to 92.7% at 300 μL L^−1^, while complete inhibition (100%) was achieved at concentrations of 500 μL L^−1^ and above. The estimated MIC_95_ value of 297.6 μL L^−1^ further confirms the high efficacy of PDEO against *R. solanacearum* race 2. These findings are consistent with previous studies reporting strong antibacterial activity of phenolic-rich essential oils such as oregano, thyme, and clove against *R. solanacearum*, with comparable MIC values [67].

The complete inhibition observed at 500 μL L^−1^ suggests that PDEO has considerable potential as a sustainable control alternative. The high efficacy at relatively low concentrations (500 μL L^−1^ with 100% inhibition) strengthens its potential applicability in tropical agricultural systems, where reducing reliance on antibiotics and synthetic agrochemicals is a priority. This approach aligns with innovative strategies that promote the use of natural products for sustainable disease management [68].

Importantly, this study represents, to our knowledge, the first report of the antibacterial activity of *P. dioica* essential oil against *Ralstonia solanacearum* race 2. The results support its potential use in the treatment of banana propagative material, particularly rhizomes, as well as for tool disinfection, phytosanitary footbaths within banana plantations, and in soil bioremediation through essential oil application or crop rotation strategies in integrated disease management programs. Overall, these findings highlight the potential of *P. dioica* essential oil as a promising candidate for the development of environmentally friendly bio-bactericides aimed at the sustainable management of Moko disease. However, additional field-scale studies are necessary to validate their safety, cost-effectiveness, and long-term efficacy before widespread application to Moko disease management.

## 4. Materials and Methods

### 4.1. Plant Material and Essential Oil Extraction

Fruits from *Pimenta dioica* were collected from small farms in Teapa, Tabasco, Mexico. *P. dioica* (Myrtaceae) is a tropical arboreal species commonly known as allspice or pimento. Allspice, also known as “pimenta gorda” is an evergreen plant with dark green leaves, and its fruits are widely used as a food condiment and in traditional medicine [69]. A voucher specimen was deposited in the herbarium of the Tropical Agriculture Garden at Chapingo University, and it can be consulted on the CICY website (CICY), access Figure 1875. After collecting, the fruits were dried in a forced-air oven at 60 °C for 72 h. The dried material was ground using a manual mill until particles of approximately 8 mm were obtained. The samples were stored in sealed flasks until the essential oil extraction.

*Pimienta dioica* essential oil (PDEO) was extracted by hydro-distillation for 1 h using a Clevenger-type apparatus [70]. Briefly, 50 g of dried plant material were placed in a flask containing 500 mL of distilled water and heated at 98 °C for 1 h. The vapor mixture was condensed, and the essential oil was collected in the graduated receiver. The obtained oil was dried over anhydrous sodium sulfate, transferred to amber glass vials, and stored at 4 °C until further analysis.

### 4.2. Chemical Characterization

The chemical composition of PDEO was analyzed by gas chromatography-mass spectrometry using an Agilent 6890 gas chromatograph coupled to an Agilent 5973 MSD mass spectrometer (J&W Scientific, Folsom, CA, USA). Separation was performed on an HP-5MS fused silica capillary column (5% phenyl-95% polydimethylsiloxane, 30 m × 0.25 mm i.d., 0.25 mm film thickness, J&W Scientific, Folsom, CA, USA). The GC/MSD system was operated under the following conditions: Injector temperature, 240 °C; transfer line temperature, 280 °C; oven temperature programmed 50 °C to 260 °C at 10 °C min^−1^; with a final hold time of 30 min, solvent delay, 2 min; A post-run was conducted at 270 °C min^−1^. Mass spectra were acquired in electron ionization (EI) mode at 70 eV, with a scan range *m*/*z* 40–400.

### 4.3. Plant Pathogenic Bacteria

**Figure 4:**
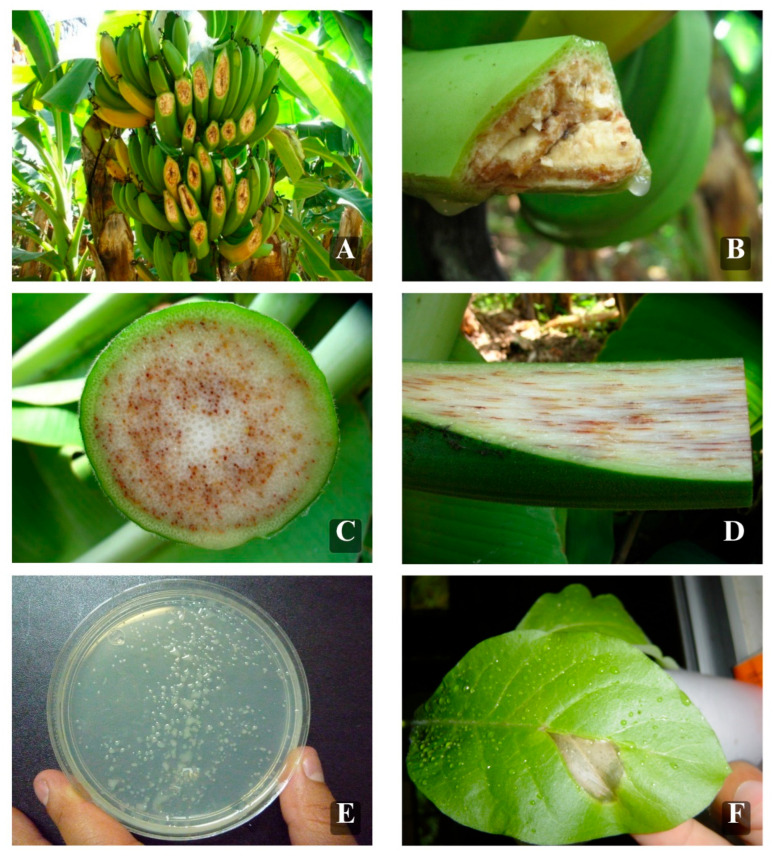
Cavendish banana plants infected with *R. solanacearum* Race 2. (**A**,**B**) Fruit symptoms; (**C**,**D**) pseudostem symptoms on vascular tissues. (**E**) Bacteria on CTZ medium. (**F**) Hypersensitivity reaction of *R. solanacearum* race 2 on tobacco leaf.

*Ralstonia solanacearum* race 2 was isolated from Cavendish banana (AAB group) plants exhibiting showing necrosis and yellowing of upper leaf, as well as vascular necrosis and brown discoloration in the pseudostem (Figure 4A–D). Samples were collected from a commercial plantation in Teapa, Tabasco, Mexico (17°31′37″ N and 92°55′43″ O, 40 masl). Under aseptic conditions, small tissue fragments were surface-disinfected and plated on potato dextrose agar (PDA), followed by incubation at 26 °C. Biochemical characterization was performed using standard methods [71]. After 48 h of incubation at 26 °C on Kelman’s tetrazolium chloride (TZC) medium, colonies appeared fluid and white with a pink center (Figure 4E). The isolates were Gram-negative rods and showed oxidation and fermentation reactions. Pathogenicity tests were conducted on vitroplants of banana to fulfill Koch’s postulates. A hypersensitivity reaction assay was performed on tobacco (*Nicotiana tabacum*) plants [72]. Leaves of four tobacco plants were inoculated with a bacterial suspension containing 2 × 10^8^ CFU mL^−1^. A hypersensitive response was observed 24 and 48 h after inoculation (Figure 4F).

The confirmed *R. solanacearum* race 2 strain was preserved at 4 °C in sterile distilled water in the microbial collection of the laboratory of Tropical Plant Pathology at Chapingo University (Oaxaca, Mexico).

### 4.4. In Vitro Evaluation of Antimicrobial Activity

The antibacterial activity of PDEO against *R. solanacearum* race 2 was evaluated using an agar dilution method. A bacterial suspension was prepared from a 48 h bacterial culture and adjusted to 1 × 10^6^ colony-forming units (CFU) mL^−1^. The concentration of the bacterial suspension was determined by preparing a series of 10-fold dilutions (10^−1^ to 10^−9^) of *Ralstonia solanacearum* race 2 cultures. Subsequently, 100 µL from each dilution was plated onto PDA medium. After incubation for 72 h at 26 °C, the colonies of *R. solanacearum* race 2 were counted.

Essential oil concentrations ranging from 100 to 1000 µL L^−1^ were prepared using 1000 µL L^−1^ of Tween 20 as an emulsifier. PDA plates amended with the corresponding concentrations of PDEO were inoculated by surface application of 100 µL L^−1^ of the bacterial suspension (1 × 10^6^ CFU mL^−1^). Control plates contained PDA without essential oil.

All the treatments were arranged in a completely randomized design with four replicates, and the experiment was conducted twice. Plates were incubated at 28 °C for 48 h. The antibacterial activity of *Pimenta dioica* essential oil was evaluated by quantifying the number of colony-forming units (CFU) of *R. solanacearum* race 2 developed on each PDA medium treatment using a Carl Zeiss microscope, Oberkochen, Germany, and microphotography analysis was performed with Axio Vision LE Rel. 4.4 digital software.

The minimum inhibitory concentration (MIC) was defined as the lowest concentration completely inhibiting visible bacterial growth. Probit analysis was performed using the SAS (PROC PROBIT) to estimate the minimum inhibitory concentration 50% (MIC_50_) and 95% (MIC_95_).

### 4.5. Determination of Antimicrobial Activity on Moko Disease Control

The experiment was arranged in a completely randomized design with five replicates per treatment. Three-month-old Cavendish banana vitroplants were carefully removed from polybags, washed with sterile water, and root-pruned to approximately 10 cm prior to inoculation.

Plants were immersed for 30 min in a bacterial suspension of *R. solanacearum* race 2 adjusted to 1 × 10^8^ CFU mL^−1^ in sterile distilled water. After inoculation, banana vitroplants were treated by immersion for 3 min in PDEO at different concentrations ranging from 300 to 5000 µL L^−1^. Untreated vitroplants served as the control. Both treated and control vitroplants were transplanted into polybags containing sterile soil and maintained under greenhouse conditions for disease evaluation.

### 4.6. Disease Index

Disease severity was recorded weekly for 42 days. Severity was assessed according to the Moko disease scale described by [20], as follows: 0 = healthy plant without symptoms; 1 = no leaf wilting but xylem vessel darkening; 2 = wilted leaves; 3 = wilted and yellow leaves; 4 = necrotic leaves with a green pseudostem; 5 = plant death (necrotic leaves and pseudostem).

### 4.7. Statistical Analysis

Data from all experiments were analyzed by using one-way analysis of variance (ANOVA) Statistical Analysis System software (SAS V. 9.10), with a significance level of 95%. When significant differences were detected, Tukey’s multiple range test was applied for mean comparisons (*p* < 0.05). MIC data were analyzed by Probit analysis using the SAS PROC PROBIT procedure to estimate the minimum inhibitory concentration required to inhibit 50% (MIC_50_) and 95% (MIC_95_) of bacterial growth.

## 5. Conclusions

The results of this study demonstrate that *Pimienta dioica* essential oils exhibit strong antibacterial activity against *Ralstonia solanacearum* race 2. On the other hand, eugenol was the main compound identified in Pimenta essential oil. This study suggests that PDEO, a novel and eco-friendly product containing a bioactive antibacterial compound, could constitute an alternative approach for prophylactic and curative treatment and contribute to the integral and sustainable management of Moko disease in plantain and banana crops.

Overall, this study reinforces the importance of exploring natural bioactive compounds as sustainable tools for controlling *R. solanacearum* race 2, contributing to reducing reliance on synthetic agrochemicals and promoting environmentally responsible banana and plantain production systems.

## Figures and Tables

**Table 1:** Chemical composition of essential oils evaluated against *Ralstonia solanacearum* race 2.

Compound	Concentration (%)	T_R_ * (min)
β-Myrcene	4.17	5.48
α-Phellandrene	1.11	5.73
Benzene, 1-methyl-2-(1-methylethyl)-	0.98	6.02
.beta.-Phellandrene	1.03	6.1
Eucalyptol	3.4	6.13
1,3,6-Octatriene, 3,7-dimethyl-, (Z)-	0.82	6.31
1,4-Cyclohexadiene, 1-methyl-4-(1-methylethyl)-	0.47	6.51
Cyclohexene, 1-methyl-4-(1-methylethylidene)-	1.39	6.96
3-Cyclohexen-1-ol, 4-methyl-1-(1-methylethyl)-	0.91	8.3
3-Cyclohexene-1-methanol, .alpha.,.alpha.,4-trimethyl-, (S)-	1.07	8.48
3-Allyl-6-methoxyphenol (Eugenol)	72.6	10.81
Cyclohexane, 1-ethenyl-1-methyl-2,4-bis(1-methylethenyl)-, [1S-(1.alpha.,2.beta.,4.beta.)]-	0.8	11.29
Caryophyllene	6.13	11.71
1,4,7,-Cycloundecatriene, 1,5,9,9-tetramethyl-, Z,Z,Z-	1.41	12.15
Eudesma-4(14),11-diene	0.75	12.57
Naphthalene,1,2,3,4,4a,5,6,8a-octahydro-4a,8-dimethyl-2-(1-methylethenyl)-, [2R-(2.alpha.,4a.alpha.,8a.beta.)]-	0.98	12.67
1H-Indene 1-ethylideneoctahydro-7a-methyl- (1E 3a.alpha. 7a.beta.)-	0.68	13.77
Azulene, 1,2,3,3a,4,5,6,7-octahydro-1,4-dimethyl-7-(1-methylethenyl)-, [1R-(1.alpha.,3a.beta.,4.alpha.,7.beta.)]	0.91	14.58
1,2-Benzenedicarboxylic acid butyl 2-methylpropyl ester	0.37	17.68

* T_R_. Retention time.

**Table 2:** Inhibitory activity of essential oil against *R. solanacearum* race 2 after 48 h of incubation in vitro.

Essential Oil	EO Concentration (µL L^−1^)/*R. solanacearum* Race 2 Inhibition of Colonies Formed Units (CFU) (%)				Pr > F
	100	300	500	1000	
*Pimenta dioica*	68.9 ^c^ *	92.7 ^b^	100 ^a^	100 ^a^	0.0001

* The values are presented as percentage inhibition of colony-forming units and correspond to the average of five repetitions, with standard error, and the experiment was repeated twice. Values in the same letter in the same arrow indicate no significant differences according to Tukey’s test (*p* < 0.05).

**Table 3:** Probit analysis to determine the minimum inhibitory concentration (MIC 50 and 95) of *R. solanacearum* race 2 to pimenta essential oil.

Essential Oil	B ± EE ^&^	MIC (µL L^−1^) *		*p* > X^2^ ***
		MIC_50_	MIC_95_	
*P. dioica*	1.81 ± 0.40	65.0 (41.0–85.0)	297.6 (235.5–436.0)	0.0001

B = value of slope; ^&^ = standard error of the slope; *** = Probability that the log-dose probit line fits to a straight line. * MIC_50_ = minimum inhibitory concentration 50; MIC_95_ = minimum inhibitory concentration 95.

## Data Availability

The original contributions presented in the study are included in the article, further inquiries can be directed to the corresponding author.
